# Challenging activity and signaling bias in tachykinin NK1 and NK2 receptors by truncated neuropeptides

**DOI:** 10.1016/j.jbc.2025.108522

**Published:** 2025-04-19

**Authors:** Jacob E. Petersen, Artem Pavlovskyi, Jesper J. Madsen, Thue W. Schwartz, Thomas M. Frimurer, Ole H. Olsen

**Affiliations:** 1 Novo Nordisk Foundation Center for Basic Metabolic Research, University of Copenhagen, Copenhagen N, Denmark; 2 Department of Molecular Medicine, Morsani College of Medicine, University of South Florida, Tampa, Florida, USA; 3 Center for Global Health and Infectious Diseases Research, Global and Planetary Health, College of Public Health, University of South Florida, Tampa, Florida, USA

**Keywords:** cross-reactivity, G protein–coupled receptor (GPCR), G_q_ and G_s_ signaling, molecular dynamics, molecular modeling, peptide interaction

## Abstract

The tachykinin receptors neurokinin 1 (NK1R) and neurokinin 2 (NK2R) are G protein–coupled receptors that bind preferentially to the natural peptide ligands substance P (SP) and neurokinin A (NKA), respectively. The peptide ligands share a common C-terminal sequence, Phe-X-Gly-Leu-Met-NH_2_, which contributes to their partial cross-reactivity with each other's nonnative receptors. This study examines the impact of truncated tachykinin SP and NKA analogs on signaling activity. SP and NKA were progressively truncated, yielding the shortest versions SP(6–11) and NKA(5–10) with free and acetylated N terminal. A total of 12 SP and 10 NKA analogs were evaluated for activity in bioluminescence resonance energy transfer-based cAMP and IP_3_ accumulation assays targeting both NK1R and NK2R, corresponding to G_s_ protein and G_q_ protein activation, respectively. As previously demonstrated, the first three amino acids are dispensable. When activated by SP analogs, NK1R favors activation of G_s_ over G_q_, though this difference diminishes with shorter analogs. In contrast, when NK1R is activated by NKA analogs, the G_q_ potency exceeds G_s_ potency by nearly an order of magnitude. For NK2R activation by NKA analogs, there are only minor differences between G_q_ and G_s_ potencies, with a slight preference for higher G_q_ potency. The N-terminal charge status plays a key role, leading to significant differences in analog potency. These findings provide valuable insight into how specific receptor-ligand interactions influence downstream G-protein signaling in G protein–coupled receptors, which are highly relevant for therapeutic applications. Finally, the proposed “message-address” model of neuropeptide signaling is assessed for NK1R and NK2R using truncated SP and NKA analogs.

Significant research efforts have been focused on exploring the neuropeptide-dependent signaling mechanisms of the neurokinin receptor (NKR) family (1, 2) and their associated peptide ligands, known as tachykinins. Within the G protein–coupled receptor (GPCR) superfamily, specifically the rhodopsin-like class (Class A), the NKR family consists of three distinct members: neurokinin 1 receptor (NK1R), NK2R, and NK3R. Each receptor is selectively activated by its own high-affinity peptide ligand—NK1R by substance P (SP), NK2R by neurokinin A (NKA), and NK3R by neurokinin B (NKB). Although these endogenous ligands differ in their N-terminal sequences, they share a conserved C-terminal sequence motif that enables them to activate any of the three NKRs. They have been associated with various conditions, including eye disorders (3), chemotherapy-induced nausea and vomiting *via* approved NK1R antagonists, anxiety disorders and asthma (4, 5, 6, 7) through NK2R antagonists, and diseases like schizophrenia, hypertension, reproductive disorders, and preeclampsia through NK3R (7). Moreover, NKRs are being investigated as potential treatments for obesity and related conditions (8, 9, 10, 11, 12). Notably, a recent breakthrough shows that NK2R agonists hold promise as candidates for obesity treatment since a long-acting NK2R agonist was shown to significantly reduce body weight, blood glucose, triglycerides, and cholesterol levels in diabetic, obese macaques, while also improving the insulin resistance (13).

Recent years have seen rapid advancements in structural knowledge of NKRs, with X-ray crystallography revealing the structures of NK1R in complex with various antagonists (14, 15, 16), paving the way for ensemble studies (17, 18, 19) and new functional insights. Additionally, cryo-electron microscopy (cryo-EM) has resolved the structures of NK1R bound to SP and signal mediators G_q_ and G_s_, NK2R bound to NKA and G_q_, as well as NK3R in complex with SP, NKB, senktide, and G_q_ (6, 7, 20, 21, 22). Interestingly, in the cryo-EM structures of NK1R bound to SP with either G_q_ or G_s_, both G-proteins adopt nearly identical conformations relative to NK1R, with only a slight difference in the positioning of the Cα5 helix of G_q_, which sits deeper than that of G_s_ (21), suggesting no preferential coupling of Gq or Gs to NK1R. However, several studies have reported biased signaling in NK1R (22, 23).

Biased signaling is fundamental to understanding ligand interactions with GPCRs, including peptide receptors. Biased signaling describes how different ligands can selectively activate specific intracellular pathways through the same receptor, favoring certain signaling mechanisms—such as G-protein activation or β-arrestin recruitment—rather than triggering pathways in a balanced fashion. This selectivity potentially enables the development of more targeted drugs, optimizing therapeutic effects while reducing side effects. The “message-address” model (24) proposes an explanation for how peptides achieve receptor selectivity. The “message” domain of the neurokinin peptide activates the receptor, while the “address” domain ensures specificity by interacting with unique receptor regions. This conceptual model suggests how structurally similar peptides can exhibit receptor subtype selectivity.

Apart from amino acid residues in the bottom of the orthosteric pocket, residues of the extracellular loops (ECLs) as well as the N terminal of the NKRs play a role in binding and specificity of their endogenous agonists (Fig. 1). Previously, it was shown in activity assays for NK2R that mainly the seven C-terminal residues of NKA (out of ten) are important for activity (20). In this study, an alanine scan and truncations of NKA (with a free N terminus) were analyzed for their activity on NK2R in activity assays. In the present work, we aim at elaborating on this observation further by testing the activity of truncated SPs and NKAs toward NK1R and NK2R in bioluminescence resonance energy transfer (BRET)-based cAMP and inositol 1,4,5-trisphosphate (IP_3_) accumulation assays. Accordingly, the two tachykinins underwent gradual truncation, yielding the shortest versions SP(6–11) and NKA(5–10). All SP and NKA analogs existed in two variants: with N-terminal acetylation (acetylated amino group: R-NH-COCH_3_, being uncharged) and with an N-terminal α−amino group (R-NH_3_^+^, being charged). Subsequently, a total of 12 SP analogs and 10 NKA analogs including acetylated full-length SP and NKA, were evaluated for activity in assays targeting both NK1R and NK2R.

**Figure 1:**
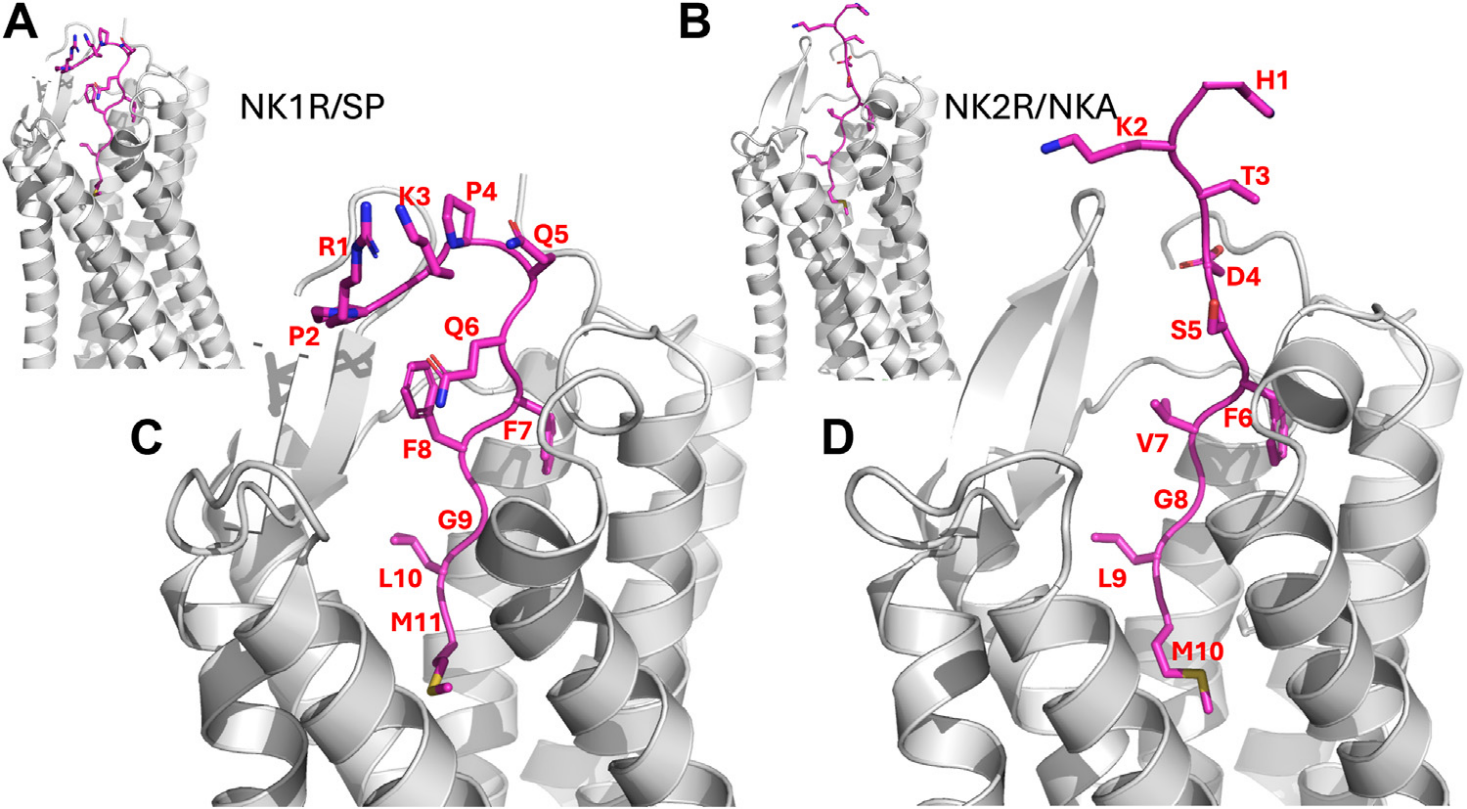
**Receptor:agonist interaction.***A*, NK1R:SP complex (PDB ID: 7p00), *B*, NK2R:NKA complex (model presented in (20)), receptors in *gray* cartoon model, agonist in *purple* stick model. *C*, NK1R:SP and *D*, NK2R:NKA, close-up of the receptor/agonist binding. SP: RPKPQQFFGLM, NKA: HKTDSFVGLM. *C*, side chains of NK1R-R177 and NK1R-Q24 (*green* stick model) form Hbond interactions to main chain carbonyls of SP-P4, SP-Q5, and SP-Q6 (*red dotted lines*). Internal Hbond between carbonyl of SP-K3 and main chain nitrogen of SP-Q6 (*blue dotted line*). *D*, side chains NK2R-D175 and NK2R-K180 (*green stick model*), and main chain nitrogen of NK2R-A25 form Hbond interactions to carboxylate of NKA-D4. Detailed agonist:receptor interaction diagrams for complexes are shown in Figure S1. NKA, neurokinin A; PDB, Protein Data Bank; SP, substance P.

The article is structured as follows. First, the activities of 10 truncated SP analogs were tested on NK1R using BRET-based cAMP and IP_3_ accumulation assays and their results were compared to those obtained for full-length SP. Interestingly, a bias toward G_s_ signaling is observed, which diminishes as the SP analogs become shorter. The shortest SP analogs, QFFGLM (SP(6–11)), exhibited a notable N-terminal characteristic: the analog with an acetylated N terminus demonstrated G_q_ activity comparable to that of SP, while the analog with a free N terminal showed activity more than an order of magnitude lower. Next, the activities of eight truncated NKA analogs were tested on NK2R and NK1R. In NK2R, the shortest analog, SFVGLM (NKA(6–10)), exhibited very low activity, while the longer acetylated NKA analogs showed a signal bias toward G_q_, with nearly identical potency, indicating that the first three positions of NKA are dispensable. The response was somewhat different in NK1R where activation by NKA analogs resulted in a bias toward the G_q_ pathway.

## Results and discussions

### Activation by SP and truncated analogs

To investigate the underpinnings of biased signaling and assess the validity of the proposed “message-address” model, a stepwise truncation approach was employed, reducing SP from SP(1–11) to SP(6–11) and NKA from NKA(1–10) to NKA(5–10). A total of 12 SP analogs and 10 NKA analogs including acetylated full-length SP and NKA, were evaluated for activity in BRET-based cAMP and IP_3_ accumulation assays targeting both NK1R and NK2R. Chemical modification of the peptides N termini was performed to determine the effect of eliminating the positively charged N-terminal α-amino group. This strategy can help clarify potential differences in the G-protein activations that are induced by various lengths of N-terminal sequences and their electrostatic contributions.

Figure 1 shows the complexes of NK1R:SP (Fig. 1, *A* and *C*, Protein Data Bank (PDB) ID:7p00) and NK2R:NKA (Fig. 1, *B* and *D*, model presented in (20)). Additionally, detailed agonist-receptor interaction diagrams for these complexes are shown in Figure S1. The conserved amidated C-terminal peptides dock into the orthosteric sites and form comparable interactions in either receptor. The subtype specificities may thus be mostly attributed to interactions between the amino acids preceding the amidated FxGLM consensus motif of the ligands and two regions of the receptor (which is the proposal of the “message-address” model): the β-hairpin in ECL2 and a segment of the N terminus leading into transmembrane (TM) helix 1 (20).

#### Activation of NK1R by SP analogs

**Figure 2:**
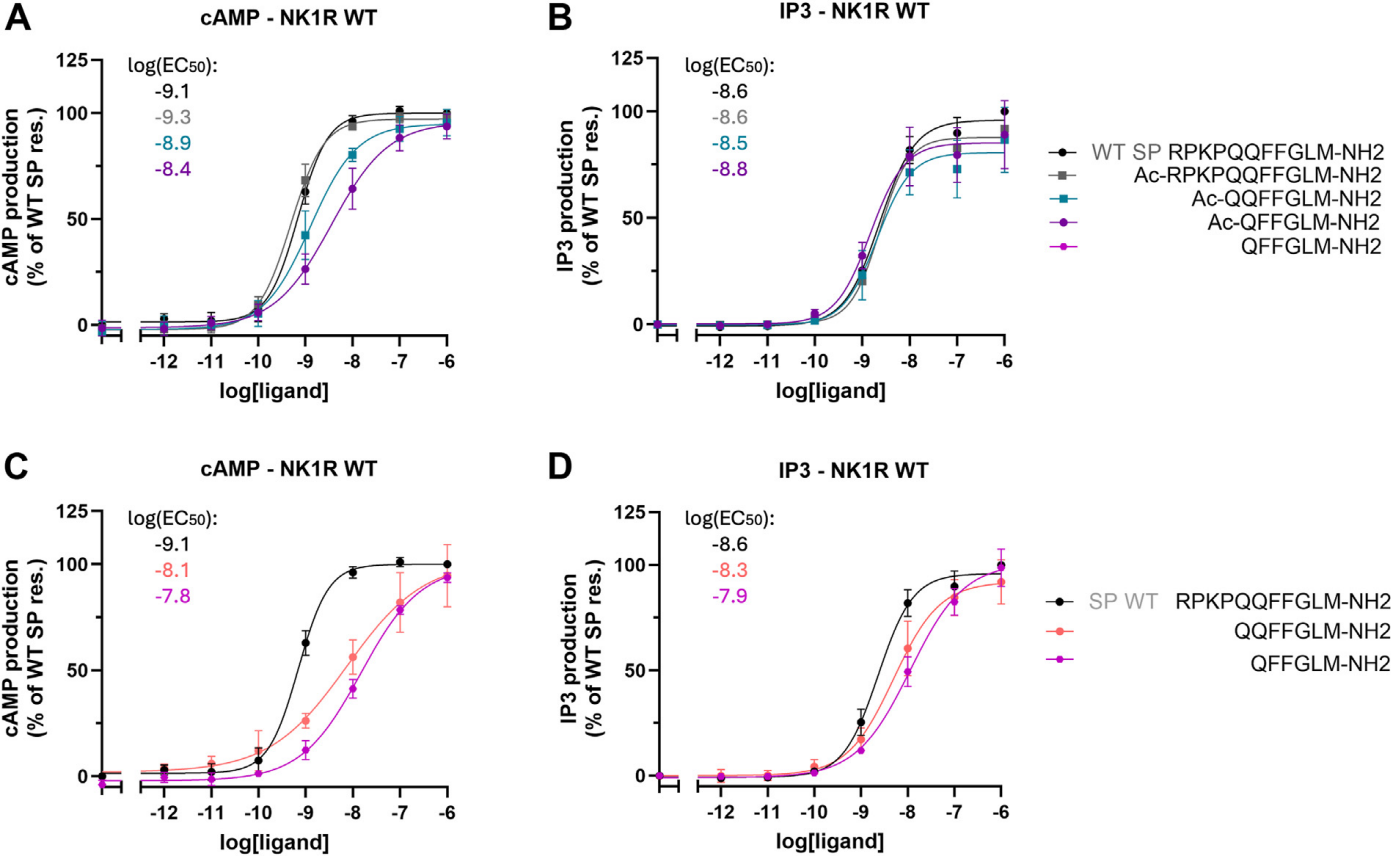
**Activation of NK1R by selected truncated SP analogs.** Results for SP (with and without acetylation), SP(5–11) (free and acetylated N termini) and SP(6–11) (free and acetylated N termini). Sequences of analogs are depicted to the *right*. To enhance the readability, the Log(EC_50_) values have been included in the figures. *A*, activation by acetylated analogs of NK1R in BRET-based cAMP assay (G_s_) and *B*, in IP_3_ accumulation assay (G_q_). *C*, activation by analogs with free N termini of NK1R in BRET-based cAMP assay and *D*, in IP_3_ accumulation assay. The difference in potency between the acetylated QFFGLM-NH_2_ and the charged analog is notable. In Table S1, the EC_50_, E_max_, error bars from functional assays corresponding to panels (*A*–*D*) are tabulated. Data represent mean ± SD from three independent experiments in duplicates. BRET, bioluminescence resonance energy transfer; NK1, neurokinin 1; SP, substance P.

Figure 2, *A* and *C* show concentration-response curves for NK1R activation by SP and selected SP analogs in BRET-based cAMP assay (G_s_ activation), while in Figure 2, *B* and *D* the results in a IP_3_ accumulation assay (G_q_ activation), are shown. In Figure S2, *C*–*F*, activity data for all peptide analogs are shown. Interestingly, the minor difference in G_s_ activation between acetylated QQFFGLM (SP(5–11)) and SP (Figs. 2 and S2, *C*–*F*) demonstrates that the first four amino acids of SP are mostly dispensable for NK1R activation by SP. These observations are also reflected in the results from the IP_3_ accumulation assay (Figs. 2*B* and S2*D*). However, in this assay, the activity deviation of the truncation compared with WT is even less pronounced. In contrast, QFFGLM (SP(6–11)) with charged N terminus has a substantially lowered activity in all experiments.

**Figure 3:**
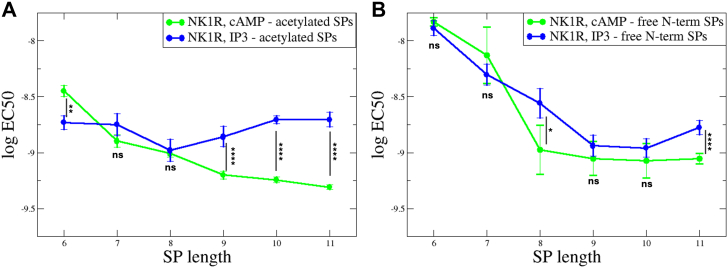
**Influence of truncated SP analogs on the potency of NK1R.** The potency, log EC_50_ versus length of truncated SPs obtained from BRET-based cAMP assay (G_s_) (*green circles*) and from IP_3_ accumulation assay (G_q_) (*blue circles*). *A*, acetylated analogs. The potency of G_q_ activation is virtually independent on length for both NK1R, while the potency of G_s_ activation decreases under the same conditions. *B*, analogs with free N-termini. Potencies for G_q_ and G_s_ decrease for shorter peptides for both NK1R. The data points are connected by lines for visual clarity. Statistical significance of differences (based on SEM estimates) between LogEC50 values was assessed using an unpaired two-tailed *t* test. Normal distribution of LogEC50 values was confirmed by the Shapiro–Wilk test. *p*-values were represented using *asterisks*: ns for *p* > 0.05 (not significant), ∗ for *p* ≤ 0.05, ∗∗ for *p* ≤ 0.01, ∗∗∗ for *p* ≤ 0.001, and ∗∗∗∗ for *p* ≤ 0.0001. BRET, bioluminescence resonance energy transfer; NK1R, neurokinin 1 receptor; SP, substance P.

To facilitate comparison of potency of analogs, the log EC_50_ values are shown in Figure 3 for NK1R activation, comparing G_q_ potency and G_s_ potency in relation to the length of the SP analogs. Figure 3*A* depicts results for activation of acetylated SP analogs on NK1R. For NK1R, the potency of G_q_ activation (blue circles) remains largely unchanged, regardless of the peptide length. This indicates a robust activation mechanism independent of truncation. Interestingly, the potency of G_s_ activation (green circles) decreases as the length of the SP analogs shortens. Additionally, the difference in potency between G_q_ and G_s_ activation—nearly an order of magnitude for full-length SP—is statistically significant but decreases for shorter SP analogs. In Figure 3*B* results for activation by SP analogs with free N-terminal α-amino group are shown. For both signaling pathways, G_q_ and G_s_, potencies drop when shorter peptides are used. However, the overall trend indicates that SP analogs promote G_s_ activation.

These observations of biased signaling for acetylated analogs are intriguing, as the cryo-EM structures of NK1R in complex with G_q_ and G_s_ reveal highly similar binding poses for SP, and the interaction regions between the intracellular portion of NK1R and both G_q_ and G_s_ are also very similar (21, 22). Therefore, a plausible explanation for the promotion of G_s_ activation by SP analogs could be their ability to enhance G_s_ binding. For NK1R, SP analogs with more than seven residues demonstrate reasonable activity, whereas peptides with six or fewer residues generally show a notable decrease in potency, except for G_q_ activation by acetylated QFFGLM (SP(6–11)). This peptide exhibits SP-like activity (Fig. 2*B*) and demonstrates significantly higher activity than its charged counterpart—an intriguing observation that will be discussed further below. Again, these data support that the first four amino acids of SP are mostly dispensable for NK1R activation, either G_q_ or G_s_, by SP.

#### Activation of NK2R by SP analogs

SP exhibits significantly lower activity toward NK2R compared to NKA. This is illustrated in Figure S2, which shows activity data from BRET-based cAMP (Fig. S2*A*) and IP3 accumulation assays (Fig. S2*B*) for SP and NKA activation of both NK2R and NK1R. In the IP3 accumulation assay, the potency of SP activation of NK2R is two orders of magnitude lower than that of the other conditions, which are nearly indistinguishable. Similarly, in the BRET-based cAMP assay, SP activation of NK2R exhibits a substantially lower potency, differing by several orders of magnitude. These observations led us to exclude activation experiments with SP analogs on NK2R, as our experience suggests that their activation would be barely measurable.

#### SP analogs binding to NK1R *versus* its activity

The observation that the first four amino acids of SP are dispensable is interesting and aligns well with the observations from cryo-EM structures of SP in complex with NK1R (21, 22). For example, in the cryo-EM structures of SP in complex with NK1R the first four side chains of SP are not resolved, probably due to their high flexibility. However, a few main chain interactions between SP and side chains of NK1R are observed. The side chain of NK1R-R177, located in ECL2, interacts with the main chain carbonyls of SP-P4 and SP-Q6, while the side chain of NK1R-Q24 (located in NK1R N-terminal) interacts with the carbonyl of SP-Q5, (Figure 1*C*). One main chain SP hydrogen bond (Hbond) from SP-K3 to SP-Q6 is observed as well. Changing the Hbond pattern by mutating NK1R-R177 to alanine or lysine reduces the binding affinity for SP by a factor of 46 and 60, respectively (21). Similarly, mutating NK1R-Q24 to alanine or asparagine reduces the binding affinity for SP by a factor of seven and five, respectively (21). The reduced binding affinity observed is modestly reflected in NK1Rs G_q_ and G_s_ activation. Given that acyl-QQFFGLM (SP(5–11)), which contains both SP-Q5 and SP-Q6, can form Hbonds with the side chains of NK1R-Q24 and NK1R-R177, only a minor shift in Log EC_50_ is observed, corresponding to a 1.1-fold enhancement in G_q_ potency and a 3.8-fold reduction in G_s_ potency. Reducing the peptide length by one more amino acid to get acyl-QFFGLM (SP(6–11)), thereby removing the Hbond to NK1R-177, changes the measured potencies remarkably little: Enhancement in G_q_ and reduction in G_s_ potencies are 1.1- and 5.0-fold, respectively for NK1R, and hence the last seven amino acids of SP are the major contributors for G_s_ activity.

**Figure 4:**
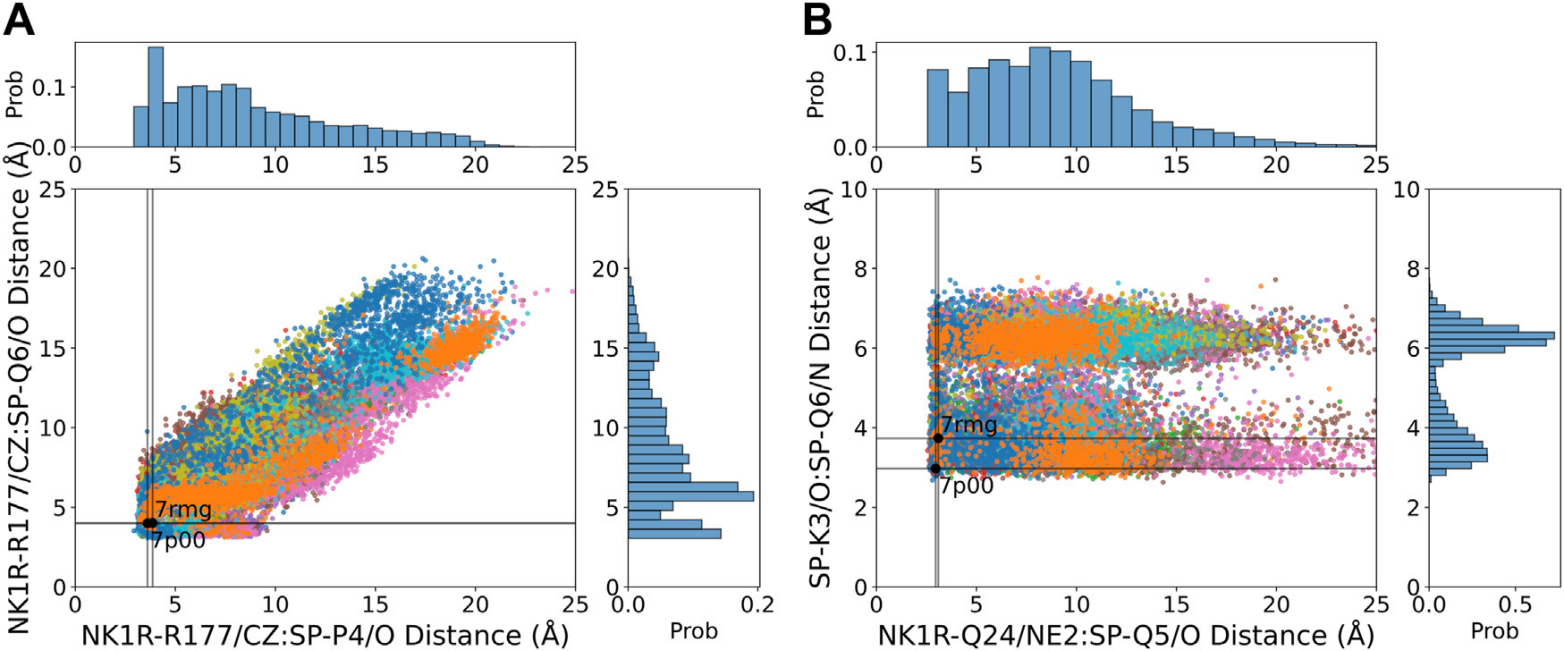
**Analyses of MD simulations of NK1R:SP complex.** Twelve trajectories of each complex taken from (22) have been overlaid (in different colors). *A*, the distances between guanidinium group of NK1R-R177CZ and the mainchain carbonyls of SP-P4 and SP-Q6. *B*, the distances between side chain of NK1R-Q24/NE2 and the carbonyl of SP-Q5/O *versus* the distance between the carbonyl SP-K3/O and the mainchain nitrogen of SP-Q6/N. Propensity histograms are depicted at the *side* and *top*. The vertical and horizontal *black lines* illustrate the spatial regions where hydrogen bonding is feasible. The histogram for SP-K3/O and SP-Q6/N interactions reveals two populations, one suggesting potential Hbond interaction. In contrast to the cryo-EM structure, very few potential Hbonds are detected for the other interactions. CHARMM atom types are used for reference. MD, molecular dynamics; NK1R, neurokinin 1 receptor; SP, substance P.

To assess the importance of the Hbonds from side chains of NK1R-R177 and NK1R-Q24 to SP as well as the internal SP Hbond, the extensive molecular dynamics (MD) simulations performed by Harris *et al.* (22) on the NK1R:SP complex were analyzed. The initial complex was taken from the cryo-EM structure (PDB ID: 7rmh) with intracellular proteins removed and subjected to 12 independent 2 μs simulations (22). The distances between the guanidinium group of NK1R-R177 and the amide of NK1R-Q24 to the mainchain carbonyls of SP-P4, SP-Q5 and SP-Q6, as well as the distance between the main chain carbonyl of SP-K3 and the mainchain nitrogen of SP-Q6 were calculated (Fig. 4). The figure includes propensity histograms at the sides and top, indicating the frequency of specific distance measurements during simulations. The vertical and horizontal black lines illustrate the spatial constraints where hydrogen bonding could occur. The histogram for SP-K3/O and SP-Q6/N distances (Fig. 4*B*) reveals two populations, the shorter of which is consistent with the Hbond interaction. In contrast to the cryo-EM structures (PDB ID: 7p00 or 7rmh) only a few potential hydrogen bond distances are detected for the other interactions. The MD simulation results suggest that a flexible N-terminal SP region disrupts the Hbonds observed in the cryo-EM structure. Thus, the apparent discrepancy or lack of correlation between the reported binding affinities and activation potencies could be explained by the possibility that, in addition to disrupting the Hbond pattern, the mutations also alter the binding cleft architecture (21).

#### Comparison of free and acetylated truncated QFFGLM (SP(6–11))

**Figure 5:**
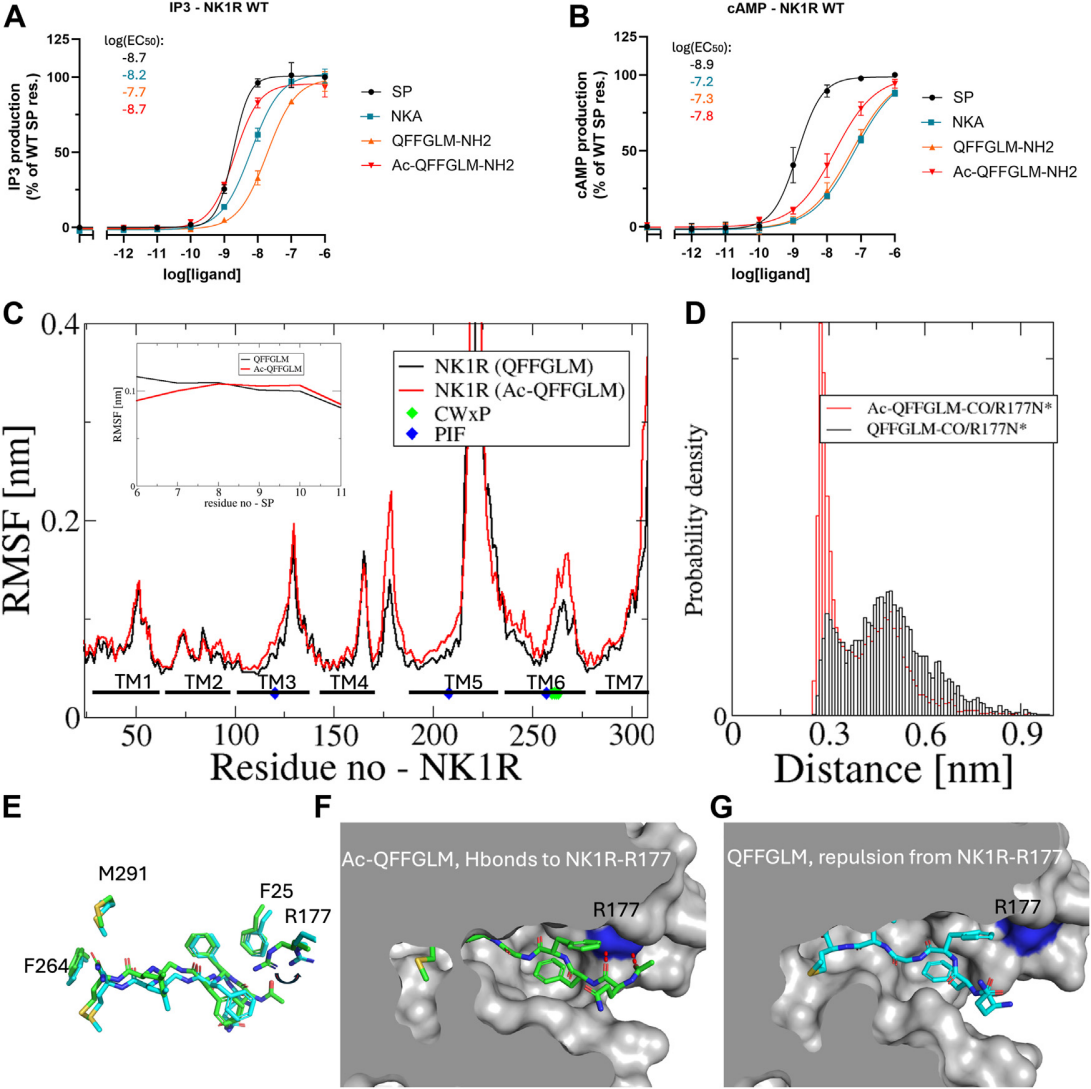
**SP(6–11) interaction with NK1R.** G_q_ activation (*A*) and G_s_ activation (*B*) by SP, NKA, as well as the acetylated and charged QFFGLM-NH_2_ analogs. Data represent mean ± SD from three independent experiments in duplicates. *C*, RMS fluctuations (for C_α_ atoms) were calculated over a 2000 ns MD simulation of NK1R:SP(6–11) complex with and without N-terminal acylation, represented by *red* and *black lines*, respectively. The location of the TM helices is indicated according to annotation in GPCRdb (35) and positions of the micro switch motifs have been indicated by *green* (CWxP) and *blue* (PIF) diamonds. TM fluctuations are generally low in the TM regions. The inset shows RMS fluctuations of SP(6–11). The acetylated N terminal of SP(6–11) exhibits less fluctuation as compared to that without acylation. *D*, Hbond probability density for the SP-Q6 main chain carbonyl (SP-Q6-CO) interaction with side chain of NK1R-R177. The elevated probability density observed for the acetylated SP(6–11) suggests an explanation for the observed reduction in the fluctuation of N-terminal C_α_. *E*, overlay of snapshots of SP(6–11) structures (acetylated (*green*) and without acylation (*cyan*)) at 250 ns in stick models. In both structures a tight aromatic ring interaction between SP-F7 and NK1R-F25 is observed. *F*, Hbond interaction between SP-Q6 and NK1-R177 in acetylated SP(6–11). *G*, without acylation, the positively charged α-amino group of SP-Q6 undergoes repulsion from NK1-R177. The presence of acylation is essential for the formation of Hbond interaction. In Table S1, the EC_50_, E_max_, error bars from functional assays corresponding to panels (*A* and *B*) are tabulated. GPCR, G protein–coupled receptor; MD, molecular dynamics; NKA, neurokinin A; NK1R, neurokinin 1 receptor; SP, substance P; TM, transmembrane.

As pointed out above, QFFGLM (SP(6–11)) with charged N terminus has a substantially lowered activity in all experiments. This analogue was extensively studied in reference (22), where it was shown to exhibit half activity in IP_3_ accumulation assay (G_q_) compared to SP, but 16-fold lower activity in BRET-based cAMP assay (G_s_). Therefore, it was claimed to demonstrate signaling biased toward G_q_. MD simulations revealed that SP(6–11) with a charged N terminus was more dynamic than SP, likely due to the absence of the N-terminal five residues (22). From Figure 2, it is apparent that acetylated QFFGLM (SP(6–11)) variant has higher affinity than the charged analog. To explain this difference in activity, MD simulations were conducted (for 2 μs) on the NK1R:QFFGLM (SP(6–11)) complex with free and acetylated N-terminal QFFGLM (SP(6–11)). The initial complexes were derived from PDB ID:7p00. Figure 5, *A* and *B* illustrate the signaling responses corresponding to G_q_ and G_s_ activation. The results of the simulations, which elaborate on the structural aspects of these activation dynamics, are presented in Figure 5*C* through Figure 5*G*.

Figure 5*A* demonstrates that the G_q_ activation potency of the acetylated analog is comparable to that of SP, maintaining similar efficacy. In contrast, the charged analog exhibits a 15-fold lower G_q_ potency. The potency of NKA lies between these two extremes, highlighting variability in G_q_ activation depending on the analog’s chemical properties. In the case of G_s_ activation (Fig. 5*B*), the results show a different trend. The activation potency of the charged analog is similar to that of NKA, indicating comparable efficacy. The acetylated analog demonstrates slightly higher activity compared to the charged analog and NKA, but its potency is still significantly lower than that of SP. This highlights how the structural modifications of the analogs influence their ability to activate the G_s_ pathway. Interestingly, the acetylated analog QFFGLM (SP(6–11)) shows a distinct activation profile toward G_q_ signaling.

The root mean square fluctuation plot in Figure 5*C* indicates that the dynamic behavior of the NK1Rs is similar. TM fluctuations are generally low in the TM regions. From the root mean square fluctuation plot of QFFGLM (SP(6–11)) (Fig. 5*C*, inset) it is observed that the C-terminal region of the acetylated version of QFFGLM (SP(6–11)) is less mobile than that of the charged QFFGLM (SP(6–11)). Further calculation of Hbond distances in terms of probability densities (Fig. 5*D*) for the interaction between the SP-Q6 main chain carbonyl (SPQ6-CO) and the side chain of NK1R-R177 (placed in ECL2) shows that acetylated QFFGLM (SP(6–11)) has a high Hbond propensity. This suggests an explanation for its reduced flexibility and enhanced activity. Figure 5*E* shows snapshots from the simulations are overlaid. The C-terminal conformations of the two QFFGLM (SP(6–11)) are very similar. However, the N-termini positions are different. The repulsion between the α-amino group of charged QFFGLM (SP(6–11)) and the side chain of NK1R-R177 (Fig. 5*G*), as well as the Hbond formation between acetylated QFFGLM (SP(6–11)) and the side chain of NK1R-R177 (Fig. 5*F*), are evident. This observation further supports the increase in enhanced activity.

### NK2R and NK1R activated by truncated NKAs

**Figure 6:**
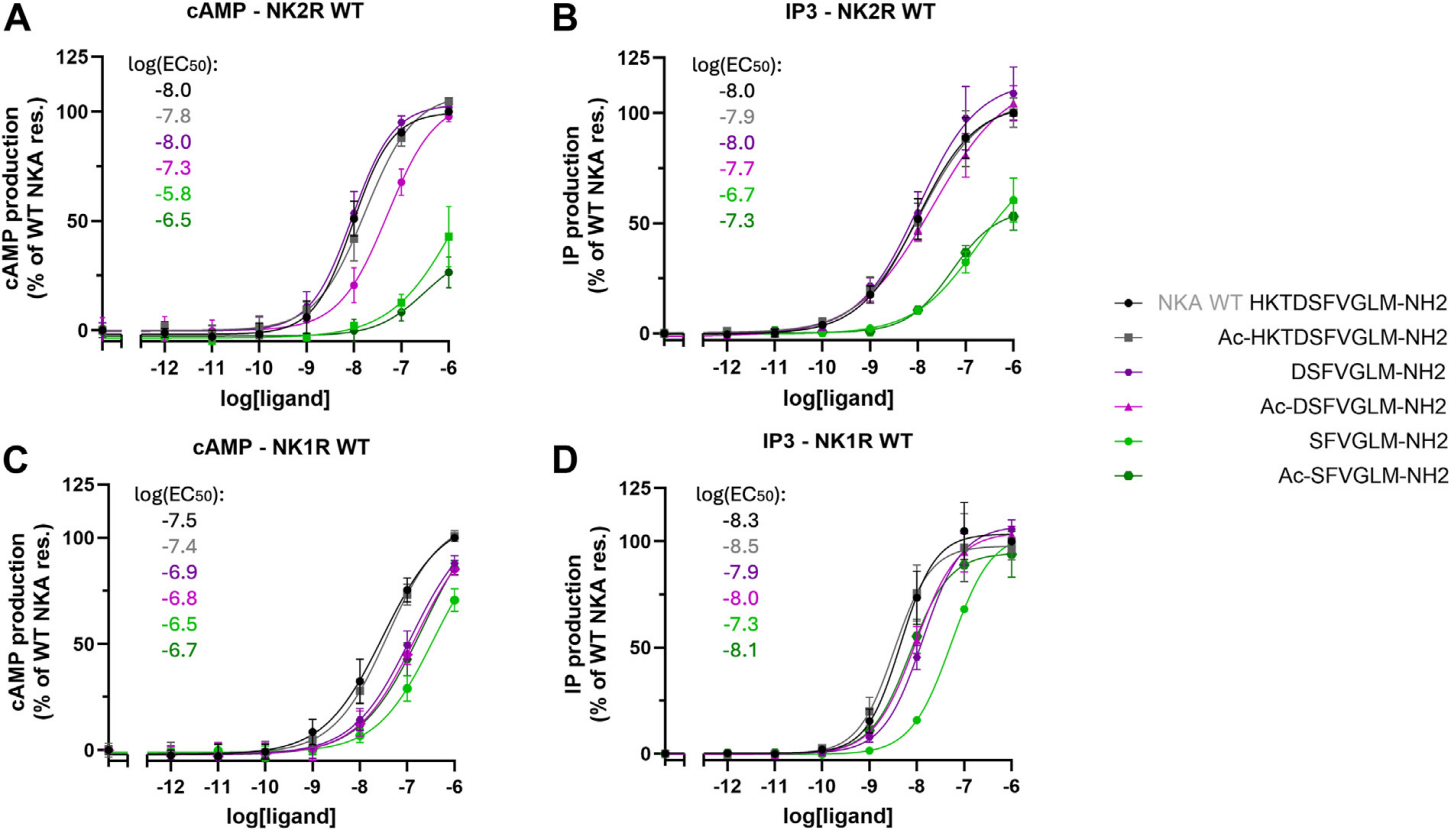
**Activation of NK2R and NK1R by selected truncated NKA analogs.** Results for NKA, and selected NKA analogs. The sequences of analogs are depicted in the middle of the figure. To enhance the readability, the Log(EC_50_) values have been included in the figures. *A*, activation of NK2R in BRET-based cAMP assay (G_s_) and *B*, activation in IP_3_ accumulation assay (G_q_). *A*, except for Ac-DSFVGLM, analogs containing NKA-D4 show activity like that of NKA while in *B*, all analogs containing NKA-D4 demonstrate activity comparable to NKA. (*A* and *B*) low activity for NKA analogs missing NKA-D4. *C*, activation of NK1R measured using the BRET-based cAMP assay, and *D*, activation measured *via* the IP_3_ accumulation assay. In both assays, SFVGLM with a free N terminal exhibits low but notable activity (*green* graphs) and shows higher potency compared to assays *A* and *B*. Additionally, the variability in activity for NKA analogs lacking NKA-D4 is greater than that observed in *A* and *B*. In Table S1, the EC_50_, E_max_, error bars from functional assays corresponding to panels (*A*–*D*) are tabulated. Data represent mean ± SD from three independent experiments in duplicates. BRET, bioluminescence resonance energy transfer; NKA, neurokinin A; NK1R, neurokinin 1 receptor.

Interestingly, SP exhibits very low affinity for NK2R (Fig. S2), whereas NKA can activate NK1R effectively (20). In Figure 6, results from activation of NK2R and NK1R by selected truncated NKA analogs (NKA, DSFVGLM (NKA(4–10)), and SFVGLM (NKA(5–10)), with free and acetylated N terminal are depicted. The corresponding sequences are provided in Figure 6. In Figure S3, results from activation of all 10 peptides are shown.

#### Activation of NK2R by NKA and truncated NKA analogs

**Figure 7:**
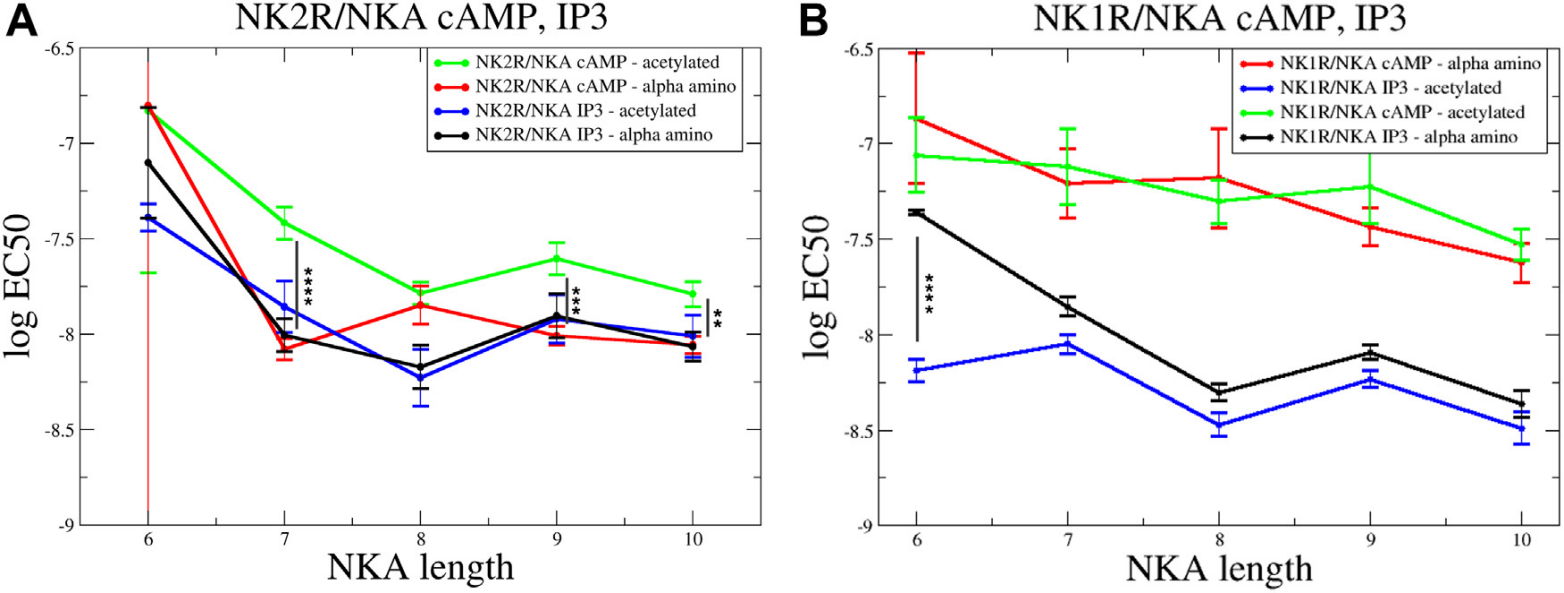
**Influence of truncated NKA analogs on the potency of NK2R and NK1R.***A*, activation of NK2R in BRET-based cAMP assay (G_s_, NKA analogs, acetylated N terminus in *green* and free N-terminus *red*) and in IP_3_ accumulation assay (G_q_, *blue*–acetylated NKA analogs and *black*–NKA analogs with free N terminus). The G_q_ activation is independent on the charge status of the N terminus while G_s_ activation by acetylated analogs is weaker but parallel compared to G_q_ activation. Apart from TDSFVGLM, the analogs with free N terminal exhibit activity level as for G_q_ activation. *B*, activation of NK1R in BRET-based cAMP assay and in IP_3_ accumulation assay, color code as in *A*. The IP_3_ data for both free and acetylated N termini exhibit the same trend, whereas for the cAMP data, the N terminus’s charge influences activation differently. Particularly, the activity of SFVGLM is influenced by a free N terminus compared to that of an acetylated. The charged N terminus of SFVGLM exhibits reduced activity. Statistical significance of differences between LogEC50 values (based on SEM estimates) was assessed using an unpaired two-tailed *t* test. Normal distribution of LogEC50 values was confirmed by the Shapiro–Wilk test. *p*-values were represented using asterisks: ns for *p* > 0.05 (not significant), ∗ for *p* ≤ 0.05, ∗∗ for *p* ≤ 0.01, ∗∗∗ for *p* ≤ 0.001, and ∗∗∗∗ for *p* ≤ 0.0001. BRET, bioluminescence resonance energy transfer; NK1R, neurokinin 1 receptor; NKA, neurokinin A; SP, substance P.

Figure 6*A* and 6*B* show NK2R activation by NKA and NKA analogs in the BRET-based cAMP assay and in the IP_3_ accumulation assay, respectively. Figure S3, *A* and *B* show that truncating the first three amino acids of NKA results in comparable activity to full-length NKA. In Figure 7*A*, the relationship between the length of NKA analogs and their activity is illustrated by plotting the log EC_50_ against the peptide length. The acetylated NKA analogs, shown as green data points, display lower G_s_ activation potency than those with a free N terminal, shown as red data points. In contrast, G_q_ activation is independent of the N-terminal's charge status (black and blue lines, Fig. 7*A*) and aligns with the trend observed in G_s_ activation by acetylated NKA analogs. However, the NKA analog of length eight (TDSFVGLM, NKA(3–10)) with a free N terminal deviates from this trend. Hence, the G_q_ activation potencies are similar regardless of the N-terminal charge status. The reduced G_s_ potency for the peptide with free N-terminal is likely due to electrostatic repulsion between its α-amino group and NK2R-K180 (Fig. 1*D*). Interestingly, when the peptide length decreases so that the α-amino group approaches the ε-nitrogen of NK2R-K180 it starts influencing G_s_ activity (see Fig. 1*D*). As shown in Figure 6*B*, DSFVGLM (NKA(4–10)) exhibits only a minor difference in G_q_ potency compared to NKA. In contrast, the difference in G_s_ potency is influenced by the N-terminal charge status (Fig. 6*A*). The analog with a free N terminal shows a five-fold increase in G_s_ potency compared to its acetylated counterpart. In both assays, the two SFVGLM analogs show very low activity independent of N terminal modification, compared to longer peptides. This highlights the importance of NKA-D4. The low impact on activity of the first three residues of NKA aligns well with previous alanine scanning results (20). Therefore, DSFVGLM (NKA(4–10)) is the shortest peptide with comparable activation potency to NKA, making it a promising starting point for developing improved NK2R agonists (25, 26). However, its similar potency in activating NK1R highlights a broader challenge, suggesting that modifications to its C terminal may be necessary for improved selectivity.

**Figure 8:**
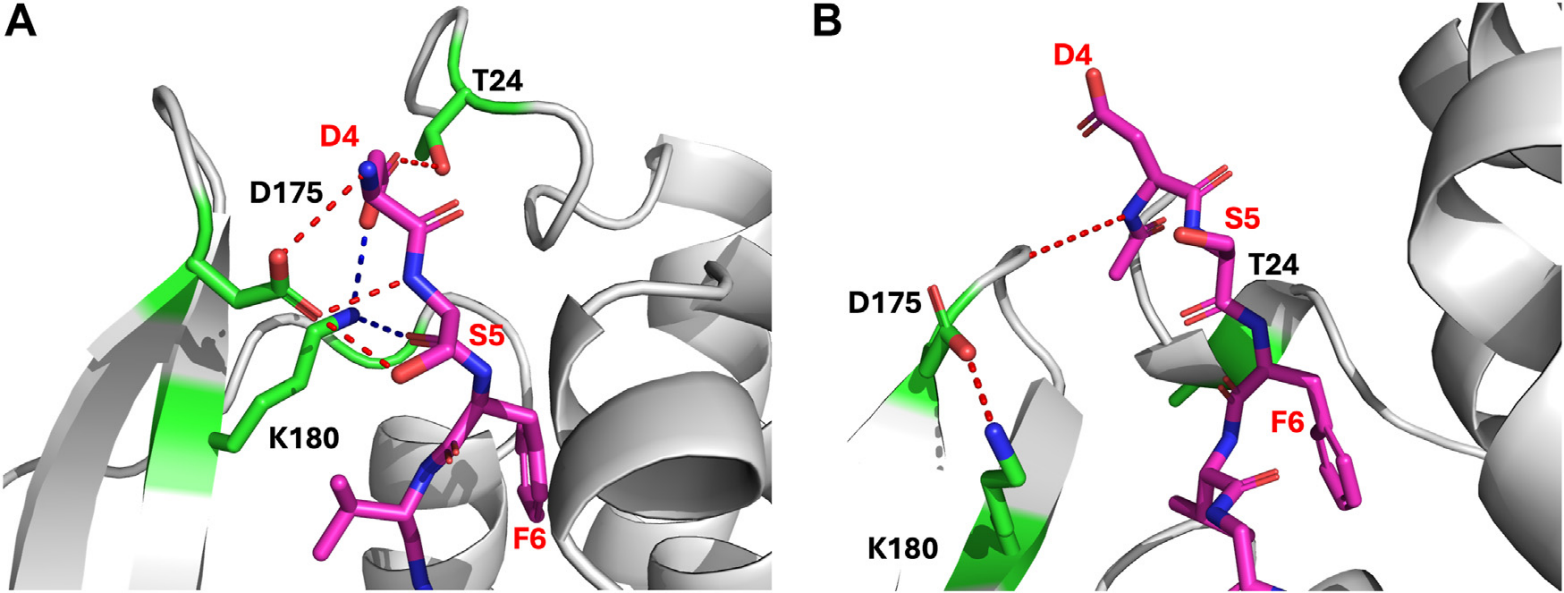
**Models of DSFVGLM (NKA(4–10) analogs bound to NK2R.** NK2R in *gray* cartoon, DSFVGLM in *purple* stick model. Close-up of Hbond interactions in complexes of DSFVGLM with free (*A*) and acetylated (*B*) N terminal. *A*, side chains interacting with DSFVGLM in *green* stick model. Hbond from carboxylate of D175 (*red* dotted lines) and ε-nitrogen of K180 (*blue* dotted lines) to DSFVGLM. D4 bridges the hydroxyl group of T24 and K180 while D175 interacts with the free N terminal of D4, hydroxyl group of S5 and its main chain nitrogen. *B*, a single Hbond from the acetylated N terminal to the *top* of ECL2 is observed. ECL, extracellular loop; NKA, neurokinin A; NK2R, neurokinin 2 receptor.

The observation of the impact of N-terminal charge status on G_s_ activation prompted us to conduct MD simulations of acetylated and free neuropeptides bound to NK2R for structural and dynamic comparisons. In Figure 8*A*, a snapshot from the simulations illustrates the tight Hbond pattern induced by the free α-amino group. Figure 8*B* illustrates how acetylation can abrogate that Hbond pattern. The impact of N-terminal modifications suggests that exploring alternative chemical modifications can introduce more pronounced activity and bias changes of possible therapeutic benefit.

#### Activation of NK1R by NKA and truncated analogs

Figure 6, *C* and *D* show NK1R activation in the BRET-based cAMP assay and the IP_3_ accumulation assay, respectively. In both assays, SFVGLM (NKA(5–10)) with a free N-terminal exhibits low but notable activity (green graphs) and shows higher potency compared to its activity toward NK2R shown in Figure 6, *A* and *B*. Additionally, the variability in activity for NKA analogs lacking NKA-D4 is greater than that observed in Figure 6, *A* and *B*. In Figure 7*B*, the relationship between NKA analogs and their activity on NK1R is illustrated by plotting the log EC_50_ against the peptide length. Apart from SFVGLM (NKA(5–10)) with a free N terminal, the G_q_ and G_s_ signaling follows the same trend independent on N-terminal charge status. The G_q_ activity of SFVGLM (NKA(5–10)) is influenced by the free N terminus when compared to that of an acetylated N terminus. The charged N terminus of SFVGLM (NKA(5–10)) exhibits reduced activity due to the electrostatic repulsion to NK1R-R177, similar to what is shown in Figure 4 for QFFGLM (SP(6–11)).

Interestingly, the activation of NK1R by SP analogs differs notably from activation by NKA analogs. Figure 3*A* highlights that acetylated SP analogs show higher potency for G_s_ activation compared to G_q_, indicating a preference for signaling *via* the G_s_ pathway. This contrasts with NK1R activation by NKA. In Figure 7*B*, it is evident that NKA analogs have improved G_q_ activation potency over that of G_s_. This suggests that NKA analogs are more effective in activating G_q_-mediated signaling pathways, potentially due to differences in receptor binding dynamics or conformational changes induced by the analogs resulting in enhanced G_q_ binding. This distinction between SP and NKA analogs activity highlights how structural variations in ligands can influence receptor signaling biases, which could be important for designing selective therapeutics.

#### Evaluating the “message-address” model applied to NK1R and NK2R

This conceptual model suggests that the neuropeptide “message” domain is the shared C-terminal sequence, Phe-X-Gly-Leu-Met-NH_2_ (for both SP and NKA), and that this domain governs their core biological activity. The “address” domains, which determine receptor specificity, are in the preceding N-terminal regions: the first six amino acids for SP and the first five for NKA. SP activates NK2R very poorly (20) due to SP-F8, which fails to appropriately complement the receptor’s orthosteric pocket. While our findings suggest that the model remains mostly true, there are important nuances to keep in mind. The “message” and “address” domains are not strictly separable, as interactions between these regions can influence receptor binding and downstream signaling. Further, the “message” domain does not appear to dictate downstream signaling bias (*e.g.*, G_q_ *versus* G_s_ pathways). Still, the model illustrates how structurally similar peptides can exhibit receptor subtype selectivity. Together, these concepts are crucial for refining drug design and enabling more precise control of GPCR activity.

## Conclusion

Previously (20), the effect of truncated NKAs with a free N-terminal and alanine-modified NKAs on NK2R was evaluated, demonstrating that the first three amino acids are dispensable. In the present work, we investigated the effects of truncated NK1R and NK2R agonists on G_q_ and G_s_ signaling pathways mediated by NK1R and NK2R. The study aimed to elucidate the structural basis of receptor-specific signaling by truncated NK1R and NK2R agonists through G_q_ and G_s_ pathways. Additionally, we sought to provide insights into minimizing cross-reactivity, which is desirable for drug design to ensure more targeted therapeutic interventions using shortened agonists.

The two tachykinins underwent gradual truncation, down to their shortest versions, SP(6–11) and NKA(5–10). All SP and NKA analogs were tested in two variants: with N-terminal acetylation (uncharged acetylated amino group: R-NH-COCH_3_) and with a free N-terminal (charged α-amino group: R-NH_3_^+^), yielding a total of 12 SP analogs and 10 NKA analogs including acetylated full-length SP and NKA to be evaluated for activity in assays targeting both NK1R and NK2R. In both cases, the first three amino acids of SP and NKA were dispensable. For shorter analogs (such as QFFGLM (SP(6–11))), the N-terminal charge status plays a significant role in the peptide-receptor interaction.

For NK1R activation by acetylated SP analogs (Fig. 3*A*), the G_s_ activation potency exceeds G_q_ potency for peptides of length 8 to 11, where the G_q_ potency remains nearly independent of peptide length. However, this difference diminishes for shorter peptides. When the peptides have a free N terminal (Fig. 3*B*), the influence of length is more pronounced, but overall, the trend mirrors that of acetylated peptides.

For NK2R activation by NKA analogs (Fig. 7*A*), there are only minor differences between G_q_ and G_s_ potencies, with a slight preference for better G_q_ potency. The potency is largely independent of the peptide’s N-terminal charge. The shortest peptide with activation potency comparable to NKA is DSFVGLM, making it a promising candidate for developing enhanced NK2R agonists. However, its similar potency in activating NK1R highlights a broader challenge, suggesting that modifications to its C terminal may be necessary for improved selectivity. For NK1R activation by NKA analogs (Fig. 7*B*), the G_q_ potency exceeds that of G_s_ by nearly an order of magnitude. Activation is independent of the peptide’s charge status, except for the shortest analog, SFVGLM (NKA(5–10)), where charge repulsion lowers the G_q_ activity.

Notably, SP analogs tend to promote G_s_ activation of NK1R, whereas NKA analogs favor G_q_ activation.

## Experimental procedures

### Structural modeling

Structural visualization was performed in PyMOL (open_source version 2.5.0, Schrödinger LLC, http://www.pymol.org/pymol).

### Molecular dynamics simulations

#### GROMACS/CHARMM36m forcefield

Input scripts for the MD simulation of the two models of NK1R:QFFGLM with free and acetylated N terminal (in complex with the helical C terminal part of G_q_) were taken from PDB ID:7p00, modeled in PyMOL and prepared in CHARMM-GUI (27, 28). The standard input generator for membrane builder (bilayer builder) was used. The position of the membrane bilayer was calculated using the PPM server (29). The system was solvated using 22,270 TIP3P waters (30) in a rectangular box (85 × 85 × 135 Å^3^). Ionic strength of 150 mM was obtained by adding Cl^-^ (60) and Na^+^ (243) ions, and the membrane was composed of DOPS lipids (192 lipids). The simulations were performed at 303 K using a Nosé-Hoover thermostat in an NPT (constant pressure using Parrinello–Rahman barostat (31) ensemble using GROMACS (32) and the CHARMM36m force field (30). The system was minimized and simulated for 2000 ns. The trajectory was analyzed using GROMACS tools and in-house scripts. Plotting was done using Grace (xmgrace; https://plasma-gate.weizmann.ac.il/Grace/) or matplotlib (33).

### Compounds

All compounds were dissolved in 100% dimethyl sulfoxide or 70% ethanol. NKA and SP were from Sigma-Aldrich (N4267 and S6883, respectively). All truncated SP and NKA analogs were from TAG Copenhagen A/S. Peptide purity exceeding 98%.

### Plasmids

All receptor constructs of WT and modified human *TACR1* and *TACR2* were inserted into the pcDNA3.1(+)-C-DYK vector (Genscript) whereas CAMYEL (34) was expressed *via* pcDNA3.1(+) vector.

### Cell culture, plating, and transfection

COS cells were chosen because they typically produce higher levels of recombinant protein and exhibit greater transfection efficiency compared to HEK cells. Additionally, COS cells are well-suited for generating stable cell lines and are sometimes more effective at expressing and secreting membrane-bound proteins. COS7 cells were maintained in Dulbecco's modified Eagle's medium 1885 with GlutaMAX supplemented with 10% fetal bovine serum (Sigma-Aldrich), 100 units/ml penicillin and 100 μg/ml streptomycin at 37 °C with 10% CO_2_. COS7 cells were tested monthly for *mycoplasma* and kept negative. COS7 cells were plated in either clear- or white- or white with clear bottom 96-well plates that were coated with poly-D-lysine (Sigma-Aldrich) 30 min prior to cell seeding (20.000 cells/well). The following day plates were transiently transfected using calcium precipitation with 200 ng/well DNA of receptor DNA and for the cAMP assays a 1000 ng/well of CAMYEL in a culture medium with the addition of 100 μM (final concentration) chloroquine. Transfection was stopped 5 h later with the addition of a fresh maintenance medium.

### BRET-based cAMP assay

The intracellular level of cAMP was monitored in real-time using BRET. This was achieved by implementing a construct consisting of a cAMP binding protein (exchange protein activated by cAMP (EPAC)) which has been flanked by a BRET pair consisting of Renilla luciferase (Rluc) and yellow fluorescent protein (YFP). This construct is called CAMYEL (cAMP sensor using YFP-Epac-Rluc) and enables cAMP production to be sensed as Epac change conformations in response to increasing levels of cAMP, ultimately resulting in a loss of BRET signal.

On the day of the assay, white 96-well plates with COS7 cells were washed twice with 100 μl/well Hank’s balanced salt solution (HBSS) (GIBCO, Life Technologies) and preincubated for 30 min at 37 °C with 85 μl HBSS. Luciferase substrate coelenterazine h (Thermo Fisher Scientific) was added to a final concentration of 5 μM, and a baseline measurement was taken after 5 min. Concentration-response curves of either NKA or SP were added, and measurements were recorded every minute for 30 min on a CLARIOstar Plus plate reader. The BRET signal was calculated as the ratio of emission intensity at 535 nm (citrine) to the emission intensity at 475 nm (luciferase). Determinations were made in minimum duplicates.

### IP_3_ accumulation assay

COS7 cells seeded in clear 96-well plates were incubated with 0.5 μCi/ml myo [3H]inositol (PerkinElmer) in 100 μl growth medium overnight following the transfection. The subsequent day cells were washed twice with 200 μl/well HBSS (GIBCO, Life Technologies) and preincubated for 5 min at 37 °C with 100 μl/well HBSS buffer supplemented with 10 mM LiCl. Ligand addition was followed by 90 min incubation at 37 °C. To stop ligand incubation, cells were lysed with 40 μl 10 mM formic acid followed by 30 min incubation on ice. Subsequently, 35 μl of the lysate was transferred to white 96-well plates together with 60 μl of 1:8 diluted YSi SPA scintillation beads (PerkinElmer). Plates were sealed and vigorously shaken for 15 min followed by 5 min centrifugation at 1500 rpm. Measurements (scintillation) were recorded on a MicroBeta (PerkinElmer) after a 4 h delay, and determinations were made in duplicates.

### Raw and normalized data

In both assay formats, the measurement window could shift between runs without affecting the overall curve shape. Emax and Emin values often shifted uniformly upward or downward, while the sigmoidal profile remained consistent, contributing to increased standard deviation. As raw signal values are relative and can vary between batches of [^3^H]-myo-inositol or coelenterazine, each experimental run was normalized individually by setting the maximum signal from SP and NKA activation to 100% and the basal activity to 0%. In Figures. S4–S6 are shown graphs displaying the raw data corresponding to the normalized results shown in Figures S2, S3, and 5, *A* and *B*.

### Statistical analysis

All concentration-response curves have been calculated using Prism 10 software (GraphPad Software, www.graphpad.com) nonlinear regression with four parameters. SD is used for concentration-response curves to illustrate variability in experimental measurements. However, when comparing LogEC50 values, standard error of the mean (SEM) is used to reflect the precision of the estimated means and facilitate statistical comparisons. This approach ensures an accurate representation of variability while maintaining appropriate statistical rigor for hypothesis testing. All data consist of two technical replicates from three biological replicates. Statistical significance of differences between LogEC50 values (based on SEM estimates) was assessed using an unpaired two-tailed *t* test. Normal distribution of LogEC50 values was confirmed by the Shapiro–Wilk test. *p*-Values were represented using asterisks: ns for *p* > 0.05 (not significant), ∗ for *p* ≤ 0.05, ∗∗ for *p* ≤ 0.01, ∗∗∗ for *p* ≤ 0.001, and ∗∗∗∗ for *p* ≤ 0.0001.

In Table S1 the EC_50_ and E_max_ values from functional assays are tabulated.

## Data availability

MD trajectories corresponding to the data presented in Figure 5 have been uploaded to Zenodo (https://doi.org/10.5281/zenodo.14512995)

## Supporting information

This article contains supporting information.

## Conflict of interest

The authors declare that they have no conflicts of interest with the contents of this article.
