# Supplementary material for: Challenging activity and signaling bias in tachykinin NK1 and NK2 receptors by truncated neuropeptides
Source: J Biol Chem. 2025 Apr 19;301(6):108522. doi: 10.1016/j.jbc.2025.108522 (PMC12145819; doi:10.1016/j.jbc.2025.108522)
Supplement: Supporting information [file mmc1.docx]

**Supporting Information**

**Challenging activity and signaling bias in tachykinin NK1 and NK2 receptors by truncated neuropeptides**

Jacob E. Petersen^1^, Artem Pavlovskyi^1^, Jesper J. Madsen^2,3^, Thue W. Schwartz^1^, Thomas M. Frimurer^1^, and Ole H. Olsen^1*^

^1^ Novo Nordisk Foundation Center for Basic Metabolic Research, University of Copenhagen, Blegdamsvej 3b, DK-2200 Copenhagen N, Denmark

^2^ Department of Molecular Medicine, Morsani College of Medicine, University of South Florida, Tampa, Florida 33612, United States of America

^3^ Center for Global Health and Infectious Diseases Research, Global and Planetary Health, College of Public Health, University of South Florida, Tampa, Florida 33612, United States of America

^*^Correspondence: Ole H. Olsen, Section for Metabolic Receptology, Novo Nordisk Foundation Center for Basic Metabolic Research, University of Copenhagen, Blegdamsvej 3b, DK-2200 Copenhagen N, Denmark. Tel.: +45 60661288. E-mail: [oho@sund.ku.dk](mailto:oho@sund.ku.dk)

This document contains three figures (Figs. S1-S3) and one table (Table S1).


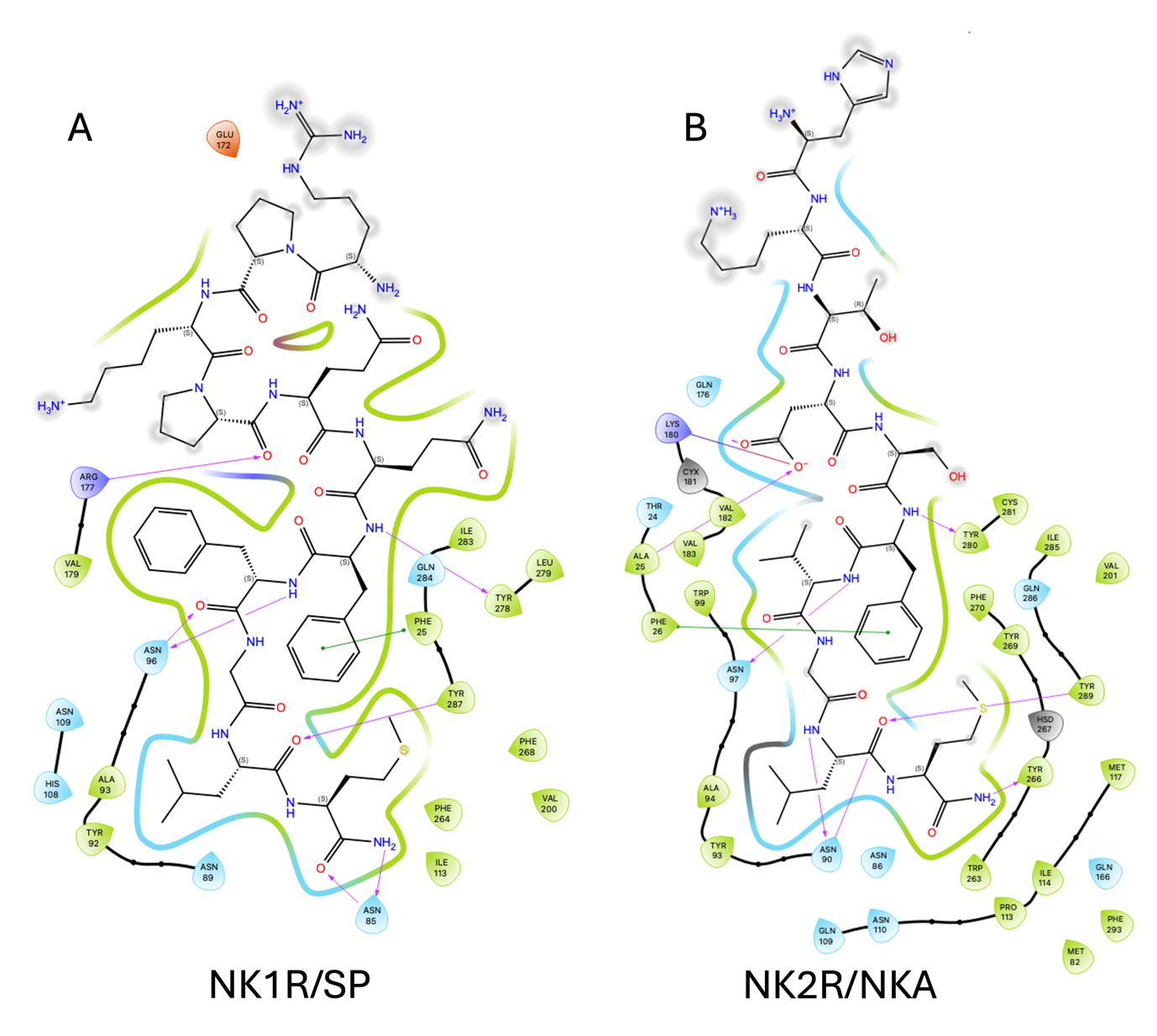


**Figure S1. Receptor/agonist interaction diagrams.** *A,* The diagram illustrates the key interactions between the agonist SP and the surrounding residues of NK1R (PDB ID: 7p00). *B,* The diagram illustrating the interaction between NKA and NK2R (structure from (20)). Hydrogen bonds are represented by red lines, hydrophobic contacts are shown as green balloons, representing non-polar interactions. Interaction with hydrophilic side chain indicated with light blue ballons, positive charged side chains indicated with dark blue balloons and negatively charged side chains with red balloons. Pi-stacking and cation-pi interactions between aromatic residues and the ligand’s aromatic rings are depicted in green lines.


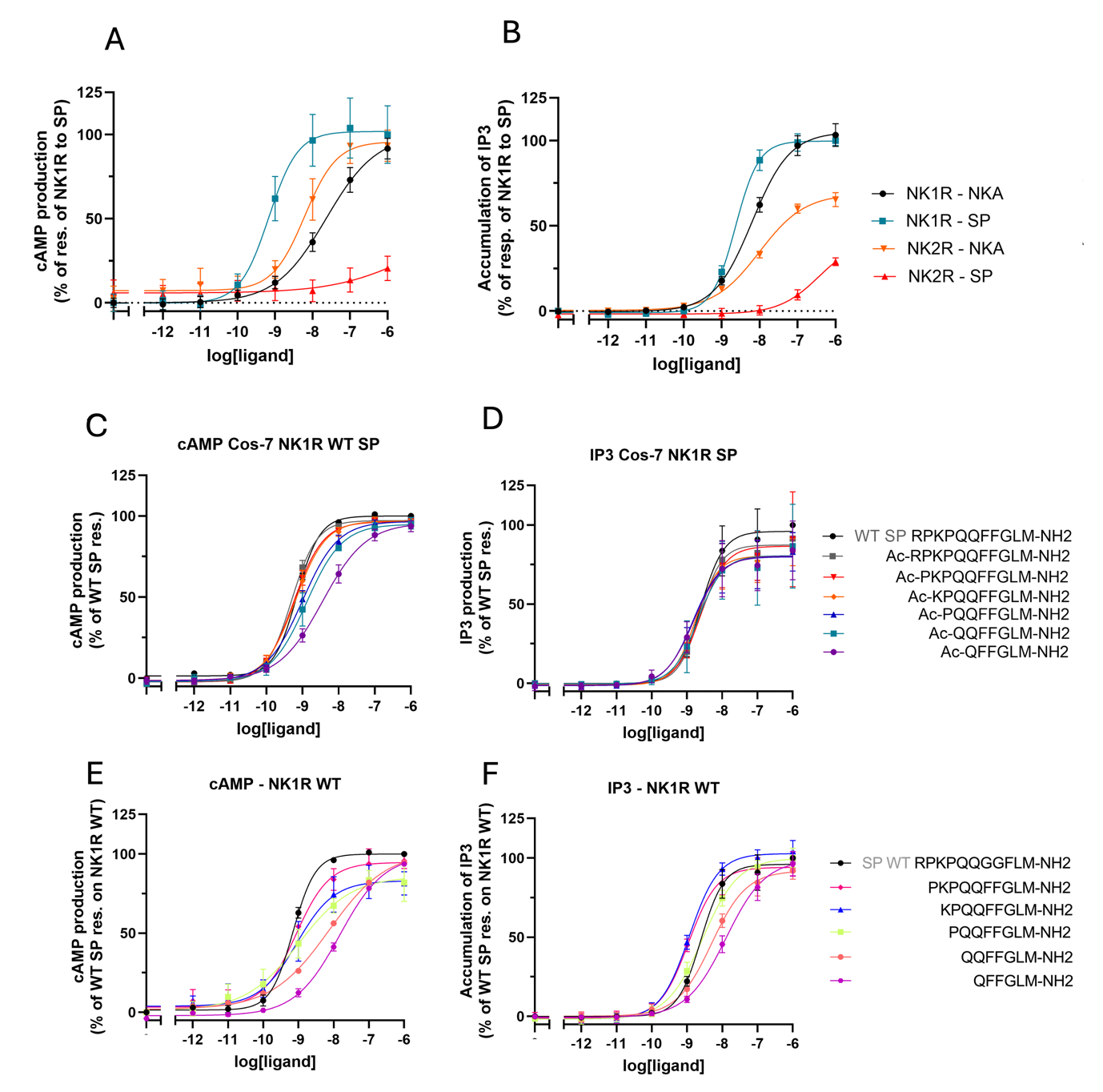


**Figure S2. Activation of NK1R and NK2R by NKA and SP (*A-B*), and activation of NK1R by truncated SP analogs (*C-F*)**. In ***A*** and ***B*** (receptor and agonist to the right), the very low activity of SP on NK2R in both assays led to an exclusion of activation experiments with SP analogs on NK2R. ***C***-***F***: Sequences of analogs are depicted to the right. Activation by acetylated analogs of NK1R in BRET-based cAMP assay (G_s_) (***C)*** and in IP_3_ accumulation assay (G_q_) (***D)***. Activation by analogs with free N-termini of NK1R in BRET-based cAMP assay (G_s_) (***E***) and in IP_3_ accumulation assay (G_q_) (***F***). In Table S1, the EC_50_, E_max_, error bars from functional assays corresponding to panels (***C***-***F****)* are tabulated.


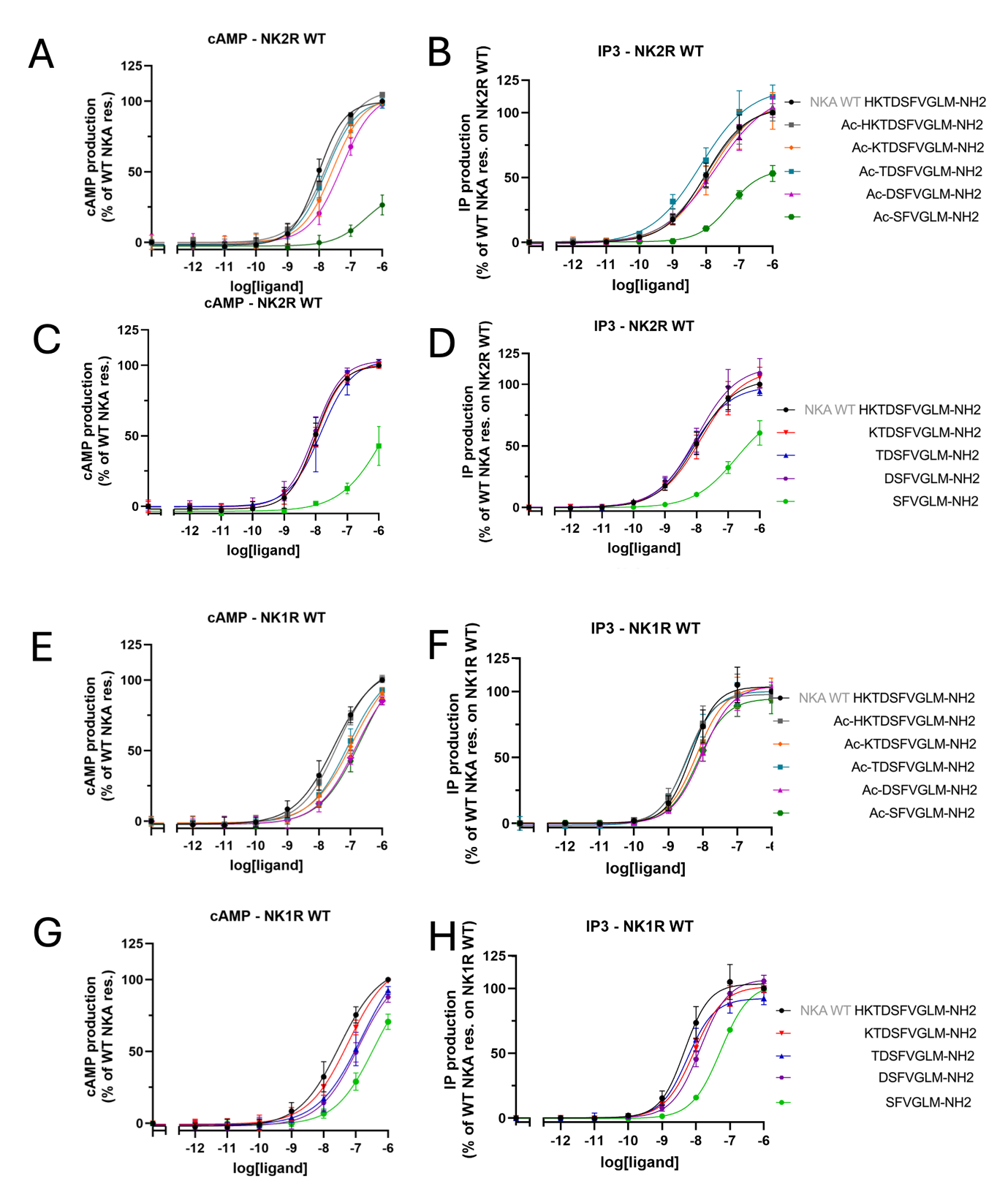


**Figure S3. Activation of NK2R and NK1R** **by truncated NKA analogs.** The sequences of the analogs are shown to the right of the figure. Activation of NK2R (***A***, ***C***) and NK1R (***E, G***) in the BRET-based cAMP assay, as well as NK2R (***B, D***) and NK1R (***F, H***) in the IP_3_ accumulation assay, are displayed. In ***A****,* ***B****,* ***E****,* and ***F***, the analogs are acetylated, whereas in ***C****,* ***D****,* ***G***, and ***H***, they have free N-termini. The analog SFVGLM (NKA(5-10)) exhibits low potency toward NK2R with or without acetylation. However, the charged analog shows low activity for NK1R activation (***G****,* ***H***), while the acetylated analog displays notable activity (***E****,* ***F***). In Table S1, the EC_50_, E_max_, error bars from functional assays corresponding to panels (***A***-***H****)* are tabulated.


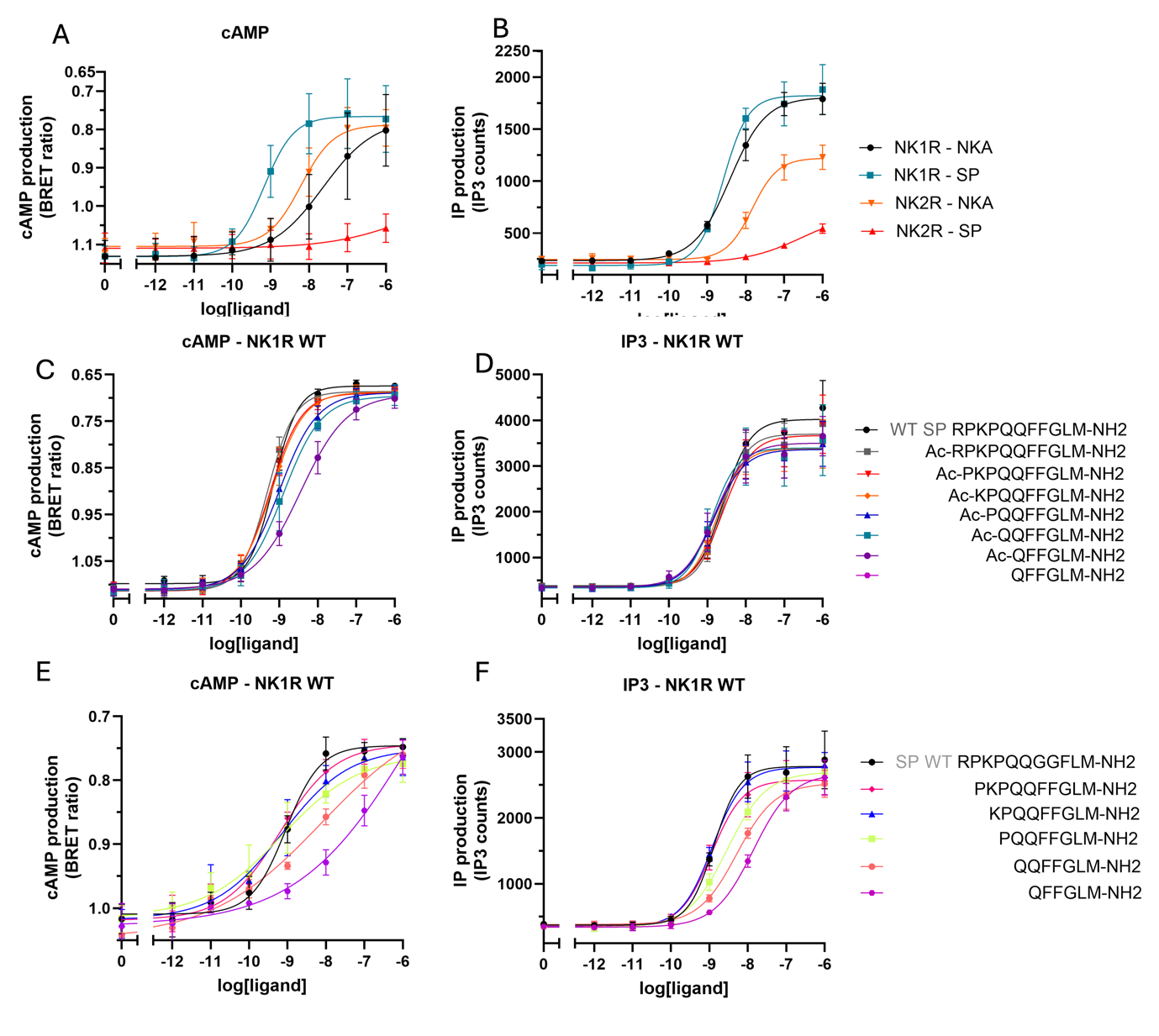


**Figure S4. Graphs displaying the raw data corresponding to the normalized results shown in Figure S2.**


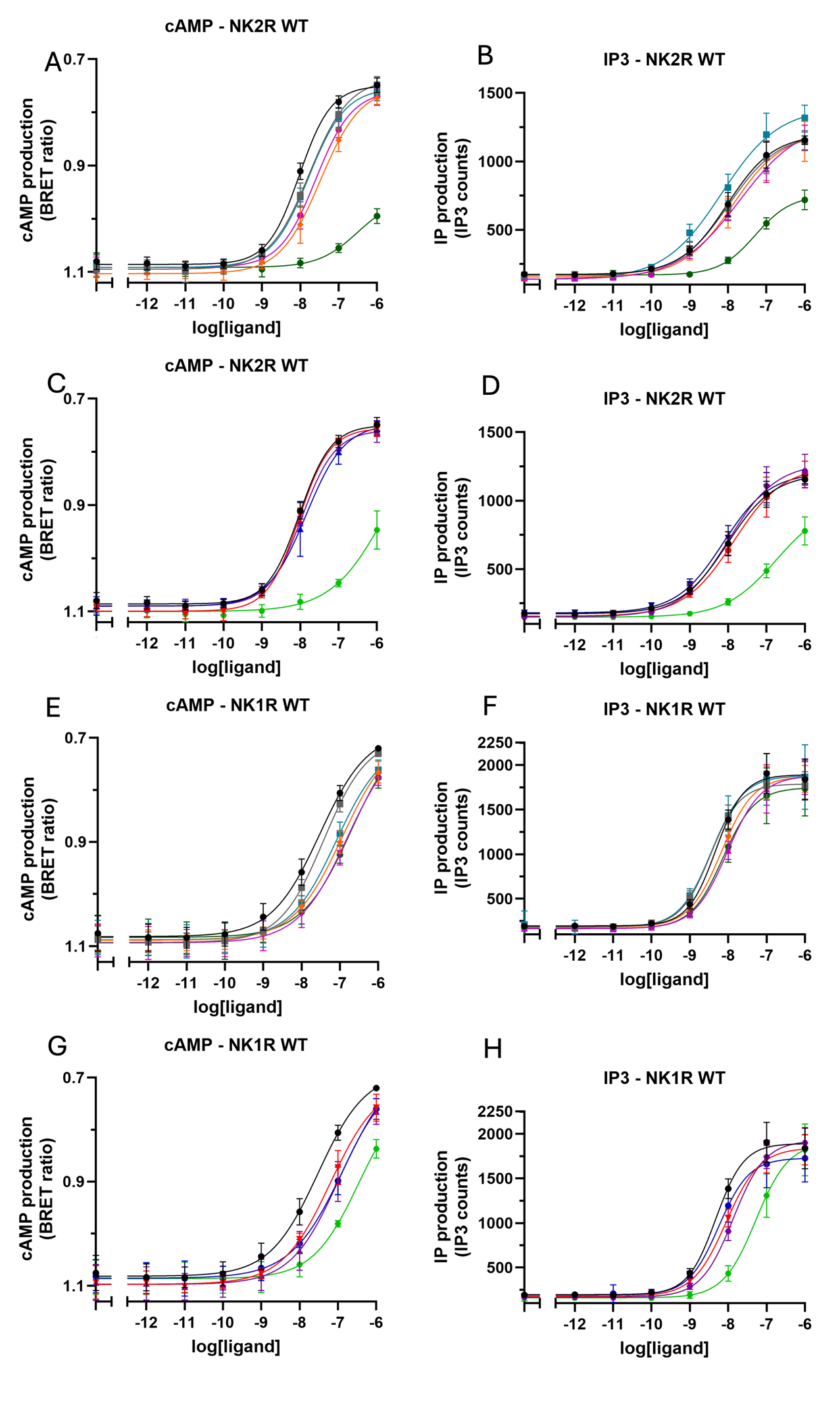


**Figure S5. Graphs displaying the raw data corresponding to the normalized results shown in Figure S3.**


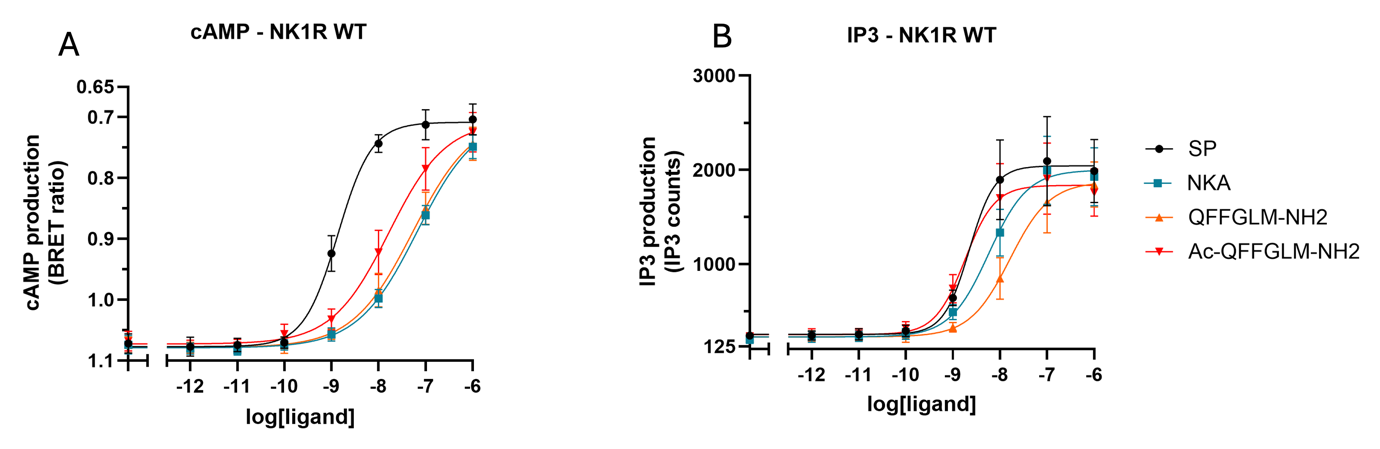


**Figure S6. Graphs displaying the raw data corresponding to the normalized results shown in Figure 5*A, B***

Table S1. **Results from BRET-based cAMP and IP_3_ accumulation assays**. In Table S1*A* are shown activation data of SP analogs on NK1R. Table S1*B* shows activation data of NKA analogs on NK1R and NK2R.
